# Mixed Matrix Membranes of Boron Icosahedron and Polymers of Intrinsic Microporosity (PIM-1) for Gas Separation

**DOI:** 10.3390/membranes8010001

**Published:** 2018-01-02

**Authors:** Muntazim Munir Khan, Sergey Shishatskiy, Volkan Filiz

**Affiliations:** Institute of Polymer Research, Helmholtz-Zentrum Geesthacht, Max-Planck-Strasse 1, 21502 Geesthacht, Germany; muntazim.khan@hzg.de (M.M.K.); sergey.shishatskiy@hzg.de (S.S.)

**Keywords:** mixed matrix membranes, polymer of intrinsic microporosity, borane, gas separation membrane

## Abstract

This work reports on the preparation and gas transport performance of mixed matrix membranes (MMMs) based on the polymer of intrinsic microporosity (PIM-1) and potassium dodecahydrododecaborate (K_2_B_12_H_12_) as inorganic particles (IPs). The effect of IP loading on the gas separation performance of these MMMs was investigated by varying the IP content (2.5, 5, 10 and 20 wt %) in a PIM-1 polymer matrix. The derived MMMs were characterized by scanning electron microscopy (SEM), thermogravimetric analysis (TGA), single gas permeation tests and sorption measurement. The PIM1/K_2_B_12_H_12_ MMMs show good dispersion of the IPs (from 2.5 to 10 wt %) in the polymer matrix. The gas permeability of PIM1/K_2_B_12_H_12_ MMMs increases as the loading of IPs increases (up to 10 wt %) without sacrificing permselectivity. The sorption isotherm in PIM-1 and PIM1/K_2_B_12_H_12_ MMMs demonstrate typical dual-mode sorption behaviors for the gases CO_2_ and CH_4_.

## 1. Introduction

Membrane technology can potentially provide environmental and economic advantages to virtually any process dependent on gas separation. However, the ability to produce durable, large-area membranes at relatively low cost and the wider application of polymeric membranes is hindered by their intrinsic permeability and selectivity limitations. These limitations were first reported by Robeson as an upper bound trade-off between permeability and selectivity and later by Freeman [1,2]. Based on the need for a more efficient membrane than purely polymeric membranes, a new concept of mixed-matrix membranes (MMMs) has been proposed. MMMs are hybrid membranes containing solid, liquid, or both solid and liquid inorganic fillers embedded in a polymer matrix [3,4]. MMMs have the potential to achieve higher selectivity with equal or higher permeability compared to existing polymer membranes while maintaining their advantages of mechanical stability and the possibility of large-scale production. Compared to pure polymer membranes, many polymer-inorganic nanocomposite membranes containing silica, carbon nanotubes, zeolite, metal organic framework (MOF), titania, etc., as IPs show higher permeability without sacrificing gas selectivity [5,6]. However, there are still many issues that need to be addressed for the large-scale industrial production of MMMs. Attempts to enhance the compatibility between the inorganic and polymeric components by introducing mutually interactive functional groups to the polymer and the molecular sieve have led to partial blockage of the sieve pores, thus hindering separation performance. 

**Figure 1:**
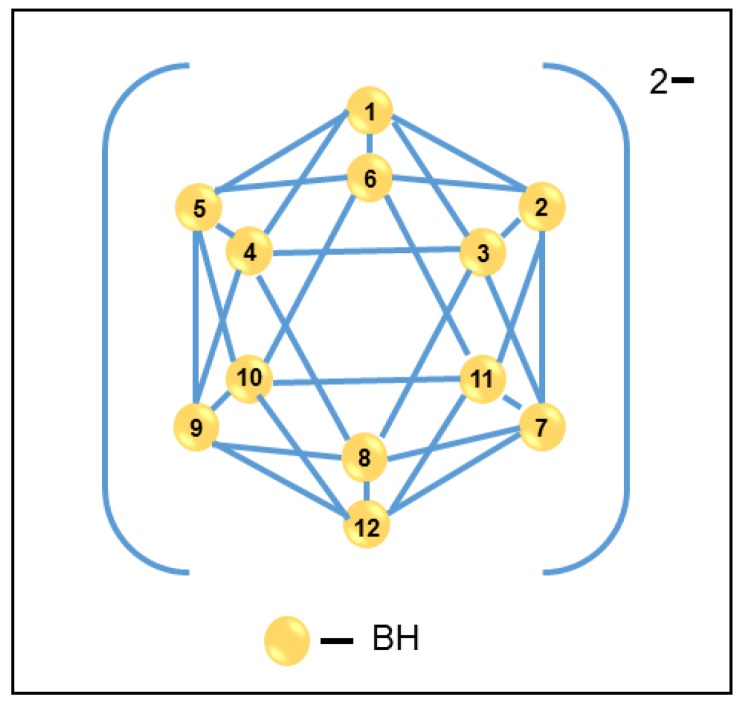
Polynuclear borane structure and numbering of atoms in the [B_12_H_12_]^2−^ anion.

Polynuclear boranes, another class of inorganic particles, have been extensively studied for the past fifty years and their chemistry is well-established and designated with the general formula B*_n_*H*_n_*^2−^ (where *n* = 6–12) [7,8]. Figure 1 shows an example of polynuclear boranes, i.e., the [B_12_H_12_]^2−^ is a dianion and bicapped square antiprism *closo* structure and B_12_H_12_^2−^ dianion has icosahedral *closo* geometry. Geometrically, polynuclear borane anions have trigonal faces. For example, icosahedral *closo*-B_12_H_12_^2−^ consists of 12 boron atoms each bonded to five neighboring boron atoms within the icosahedron and to an external atom such as hydrogen. One or more BH vertices can be exchanged for isoelectronic CH^+^ vertices, giving rise to a variety of carborane structures. Diverse functionalizations at the resulting CH vertices provide novel structures with unique applications in material science and biomedicine [9,10,11,12]. 

**Figure 2:**
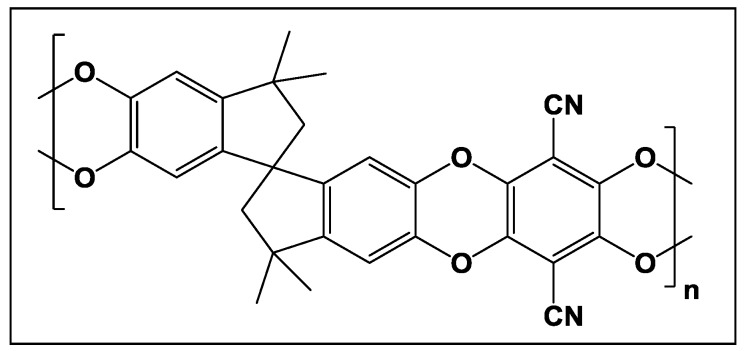
PIM-1 polymer structure.

Tailoring free volume cavities by controlling the molecular weight and the structure of glassy polymers directly influences the gas transport properties [13]. In particular, a class of high free volume polymers were potential candidates for gas separation applications with the capability to optimize gas permeability and selectivity by changing the polymer chain packing. McKeown and Budd first reported a new class of rigid ladder-type polymers containing highly contorted chains and named them polymers of intrinsic microporosity (PIM) [14]. Among these materials, PIM-1 (Figure 2), containing the contorted angled spirobisindane unit and rigid polymer backbone and high free volume, which attracted the most attention due to the combination of outstanding permeability with relatively moderate but technically attractive permselectivity, especially for O_2_/N_2_ and CO_2_/CH_4_ pairs [15,16,17].

In the present work, MMMs were fabricated by the incorporation of K_2_B_12_H_12_ (as inorganic particles) into a PIM-1 matrix (as a polymer matrix). Pure gas permeability data (H_2_, N_2_, O_2_, CO_2_ and CH_4_ gases) were reported for pristine PIM-1 and their MMMs. Physical properties such as the thermal analysis and morphology of the IPs were investigated. The prepared MMMs were characterized by scanning electron microscopy (SEM), thermogravimetric analysis (TGA), single gas permeation tests and gas sorption measurement. To the best of our knowledge, so far there is no MMM publication available on using boron icosahedron B*_n_*H*_n_*^2−^ (as an IP) combined with PIM-1 (as a polymer matrix) for a gas separation membrane. 

## 2. Theory and Background

### 2.1. Gas Sorption

In order to understand the gas transport properties of MMMs, two aspects need to be considered. First, static sorption experiments can reveal the maximum sorption capacity of a polymer for certain gas, which helps us to understand why IPs can enhance the performance of MMMs compared to pristine polymer membranes. Second, dynamic sorption experiments reveal information on the kinetics of the gas sorption from which diffusion coefficients can be determined.

#### 2.1.1. Static Gas Sorption

Gas sorption in glassy polymeric membranes described by the dual-mode sorption model. In this model, penetrant molecules are viewed as being partitioned between the dense equilibrium structure of the polymer (dissolved mode) and the non-equilibrium excess volume of the glassy polymer (the so-called hole filling or Langmuir mode) [18]. The dual mode model is described by Equation (1):

(1) $$C=C_{D}+\;C_{H}$$

where *C* is the total concentration of penetrant in the polymer (mol/g), *C_D_* is the dissolved mode penetrant concentration, and *C_H_* is the penetrant concentration in the hole filling of Langmuir mode. *C_D_* is written as a linear function of pressure and *C_H_* is expressed by a Langmuir isotherm to give:

(2) $$C=k_{D}p+\left(\frac{C\prime_{H}b_{P}}{1+b_{P}}\right)$$

where *k_D_* is Henry′s law/dissolved mode sorption constant [mol/(g·bar)], *p* the pressure (bar), *C*′*_H_* is the Langmuir/hole filling capacity constant (mol/g) and *b* is the Langmuir affinity parameter (1/bar). The parameter *k_D_* shows the penetrant dissolved in the polymer matrix at equilibrium and *b* characterizes the sorption affinity for a specific gas–polymer system. These parameters can be determined from the measured sorption data. *C*′*_H_* is often used to measure the amount of non-equilibrium excess free volume in the glassy state [19].

#### 2.1.2. Dynamic Gas Sorption

Diffusion coefficients can be accurately determined from the mass uptake curves (*M_t_*/*M_∞_*) by data-fitting Fick′s second law for the sorption of penetrant in the film as described by Crank [20]:

(3) $$\frac{M_{t}}{M_{\infty }}=1-\frac{8}{\pi }\overset{\infty }{\underset{n=0}{\sum }}\frac{1}{{\left(2n+1\right)}^{2}}exp\left[-\frac{D{\left(2n+1\right)}^{2\;}\pi^{2\;}t}{l^{2}}\right]$$

where *M_t_* and *M*_∞_ represent the amount of gas absorbed by the membrane film at time *t* and the equilibrium sorption after infinite time, respectively. *D* is the kinetic (transport) diffusion coefficient, *t* is the time required to attain *M_t_* and *l* is the thickness of the sample.

### 2.2. Gas Permeation

Gas permeation through a dense membrane takes place according to the well-known solution–diffusion mechanism [21]:

(4) $$P_{i}=S_{i}\times D_{i}$$

where the permeability coefficient (*P_i_*) in Barrer (1Barrer = 10^−10^ cm^3^(STP)·cm/(cm^2^·s·cmHg)) is the product of the solubility coefficient (*S_i_*) (cm^3^(STP)/(cm^3^·cmHg)) and the diffusion coefficient (*D_i_*) (cm^2^/s) of component *i*. The ideal selectivity for a gas pair is the ratio of their permeability coefficients:

(5) $$\alpha_{ij}=\frac{P_{i}}{P_{j}}=\frac{S_{i}\times D_{i}}{S_{j}\times D_{j}}=\left(\frac{S_{i}}{S_{j}}\right)\times \left(\frac{D_{i}}{D_{j}}\right)$$

where *D_i_*/*D_j_* is the diffusion selectivity and *S_i_*/*S_j_* is the solubility selectivity of components *i* and *j*, respectively. Diffusion coefficients increase with a decrease in the penetrant size, increasing the polymer fractional free volume, increasing polymer chain flexibility, increasing the temperature and decreasing polymer–penetrant interactions [22]. On the other hand, solubility coefficients increase with increasing polymer–penetrant interactions, decreasing temperature and the increasing condensability of the penetrant.

## 3. Materials 

The monomer 5,5′,6,6′-tetrahydroxy-3,3,3′,3′′-tetramethyl-1,1′-spirobisindane (TTSBI, 97%) was supplied by ABCR, Karlsruhe, Germany and 2,3,5,6-tetrafluoroterephthalonitrile (TFTPN, 99%) was kindly donated by Lanxess (Cologne, Germany). TFTPN was sublimated twice under vacuum prior to use. Potassium carbonate (K_2_CO_3_ > 99.5%) was dried overnight under vacuum at 120 °C in order to ensure no moisture is trapped in it and then milled in a ball mill for 15 min. Potassium dodecahydrododecaborate hydrate (K_2_B_12_H_12_·XH_2_O > 98%) was obtained from Strem chemicals Inc. (Kehl, Germany) and bis-tetrabutylammonium *closo*-dodecahydrododecaborate [N(C_4_H_9_)_4_]_2_B_12_H_12_ was supplied by Technical University Darmstadt, Inorganic solid state chemistry department. Diethylbenzene (isomeric mixture) was purchased from Sigma-Aldrich (Steinheim, Germany), dimethylacetamide (DMAc > 99%), tetrahydrofuran (THF > 99.9%), methanol (MeOH > 99.9%), chloroform (CHCl_3_ > 99.99%), dioxane (>99%), from Merck (Darmstadt, Germany) were used as received.

## 4. Experimental Section

### 4.1. Pristine PIM-1 Synthesis and Mixed Matrix Membranes Preparation

PIM-1 was synthesized by using the method described elsewhere [23,24,25,26,27]. PIM-1 and K_2_B_12_H_12_ were dried in a vacuum oven at 120 °C overnight before use. The pristine PIM-1 membrane was prepared by mixing 2% (*w*/*w*) polymer in chloroform as a solvent. MMMs were prepared with K_2_B_12_H_12_ with different weight ratios (2.5 wt %; 5 wt %; 10 wt %; 20 wt %) as determined by Equation (6).

(6) $$IPs\;loading\;=\frac{wt.\;IP\;}{wt.\;IP+wt\;polymer\;}\times 100$$

Considering a PIM-1 and K_2_B_12_H_12_ density and assuming volumes are additive, the IPs volume fraction (*φ_IP_*) were calculated according to Equation (7).

(7) φIP=wIPρIP wPρP +wIPρIP 

where *w_IP_* and *w_P_* denote the weight of IPs and polymer, respectively, and *ρ_IP_* and *ρ_P_* are the density of IPs and polymer, respectively. For the MMMs fabrication, the K_2_B_12_H_12_ was dispersed in chloroform by sonication using an ultrasonic bath (Bendelin, SONOREX Super, Bendelin Electronic GmbH & Co., KG, Berlin, Germany) for 15 min. PIM-1 was dissolved in chloroform and added to a K_2_B_12_H_12_ suspension. The resulting solution was stirred with a magnetic bar for a minimum of 15 h, and up to 60 h for a higher loading of IPs. The solution was poured into a leveled circular Teflon^®^ dish, which was covered with glass lead to reduce the chloroform evaporation rate. The slow evaporation of chloroform was ensured by 10 mL/min nitrogen flow through the closed space above the Teflon dish. After solvent evaporation, the prepared membranes were delaminated from the Teflon^®^ surface and conditioned by soaking in methanol for approximately 4 h. Immersing the membranes in methanol reverses prior to film formation history, in a manner similar to protocols previously developed for high free volume polyacetylenes and PIM-1 [28,29]. The methanol-treated membranes were dried in high vacuum for 16 h at 120 °C. The thickness of the membranes was measured by a digital micrometer (Deltascopes MP2C, Helmut Fischer GmbH, Sindelfingen, Germany), ranged between 95 to 101 µm. 

### 4.2. Thermal Gravimetric Analysis (TGA)

Investigation of the thermal stability of the pristine PIM-1, K_2_B_12_H_12_, and PIM1/K_2_B_12_H_12_ MMMs were performed by thermogravimetrical analysis (TGA) on a TG209 F1-Iris instrument from the Netzsch Company (Gerätebau GmbH, Selb, Germany). At least 10 mg of each sample was placed into a sample holder. The experiments were conducted under argon flow (20 mL/min) from 30 to 900 °C with at heating rate 10 K/min. 

### 4.3. Scanning Electron Microscopy (SEM)

A LEO 1550VP instrument (Carl Zeiss AG, Oberkochen, Germany) was used to study the morphology of pure PIM-1 and PIM1/K_2_B_12_H_12_ MMMs, which was equipped with a field emission cathode operated at 1–1.5 kV. Samples for scanning electron microscopy (SEM, Carl Zeiss AG, Oberkochen, Germany) were prepared by freezing the prepared membranes in liquid nitrogen and then breaking them to investigate the homogeneity of the IPs throughout the MMMs and compatibility between the IPs and the polymer phase. The samples were dried overnight in a vacuum oven at 30 °C and then coated with a thin Pt layer using a sputtering device under argon flow. 

### 4.4. Density Measurements

The density of the membranes was determined by the buoyancy method following Equation (8)

(8) $$\rho =\left(\frac{W_{A}}{W_{A}-\;W_{L}}\right)\rho_{L}$$

where *ρ* and *ρ_L_* are the densities of the membranes and perfluorinated liquid (Fluorinert FC 77), respectively, *W_A_* and *W_L_* are the weight of membranes in the air and in perfluorinated liquid, respectively. All the density measurements were done at 26 °C.

### 4.5. Gas Transport Properties

The permeability of single gases (H_2_, O_2_, N_2_, CH_4_, and CO_2_) were measured using a constant volume variable pressure time lag apparatus at 30 °C. The permeability (*P*), diffusivity (*D*), solubility (*S*) and selectivity (*α*) for gases *i* and *j* were determined under steady state by the following Equations [30,31,32]:

(9) P=D×S=Vpl(pp2−pp1)ARTΔt[pf−(pp2+pp12)]

(10) $$D=\frac{l^{2}}{6\theta }$$

(11) $$\alpha_{ij}=\frac{P_{i}}{P_{j}}=\frac{S_{i}\times D_{i}}{S_{j}\times D_{j}}$$

where *Vp* is the constant permeate volume, *R* the gas constant, *l* the film thickness, *A* is the effective area of the membrane, Δ*t* is the time for the permeate pressure increase from *p_p_*_1_ to *p_p_*_2_, *p_f_* is the feed pressure, and *θ* is the time-lag. The solution–diffusion transport model [21] was applied to discuss the gas transport properties of PIM-1 and PIM-1 MMMs, and the selectivities of membranes for gas “*i*” relative to another one “*j*”, which is the ratio of their permeabilities determined using Equation (6).

### 4.6. Gas Sorption

Static and dynamic sorption measurements were performed on a magnetic suspension balance (MSB) (Rubotherm GmbH, Bochum, Germany). Static sorption measurements allow the determination of the sorption isotherms, Langmuir hole affinity parameter (*b*) and the capacity parameter (*C*′*_H_*) for pristine PIM-1 and PIM1/K_2_B_12_H_12_ MMMs according to Equation (2) [19]. Dynamic sorption measurements can be used to determine the diffusion coefficient of gas in pristine PIM-1 and PIM1/K_2_B_12_H_12_ MMMs by means of Equation (3) [33].

#### 4.6.1. Static Sorption Experiments

The amount of pure gases adsorbed *m_ADS_* in the samples (PIM1/K_2_B_12_H_12_ MMMs) was calculated from the volume of the samples (calculated from the density of the samples as determined from the standard buoyancy technique explained in the above section),the gas mass uptake of the samples, and the molar volume and molecular weight of the gas probe. A minimum of 50 mg of sample was used. For each measurement, the samples were evacuated at 353 K for 18 h at *P* ≤ 10^−6^ millibar. All tubing and chambers were also degassed by applying vacuum (*P* ≤ 10^−6^ millibar).The evacuated samples were then cooled down to the specified temperature (303 K) with a ramping rate of 1 K/min. The different used gases have a purity of 99.99% in this measurement. The gravimetric sorption studies in this research were conducted at a temperature of 303 ± 0.1 K and a pressure range of 0.01–8 bar.

#### 4.6.2. Dynamic Sorption Experiments

The diffusion coefficient of gas was calculated using a dynamic sorption experiment for PIM-1 and PIM1/K_2_B_12_H_12_ MMMs. Before the start of each experiment, the thickness of the membrane samples was measured. Prior to pressurization at 1 bar, the sample was evacuated for 18 h. The mass uptake of the sample (*M_t_*) was calculated according to Equation (12):

(12) $$M_{t}=w_{t}-\left[w_{0}-\left(v_{t}\times \rho_{gas}\right)\right]$$

where *w*_0_ (g) is the weight of the sample at zero sorption, *v_t_* (cm^3^) is the volume of the sample at time *t*(s) and *ρ_gas_* is the density of the gas (g/cm^3^). To correct the recorded weight (*w_t_* (g)) for buoyancy effects, the Archimedes principle was used. Subsequently, the ratio of *M_t_*/*M_∞_* was obtained as a function of time(s). Since in the case of membranes, complete equilibrium could not be established within the time scale of the experiment, in that case, the pseudo-infinite mass uptake after 14 h was used. The obtained data were fitted using Equation (3) to obtain the diffusion coefficients for the membrane samples. 

## 5. Results and Discussion

### 5.1. Inorganic Particle Characterization

**Figure 3:**
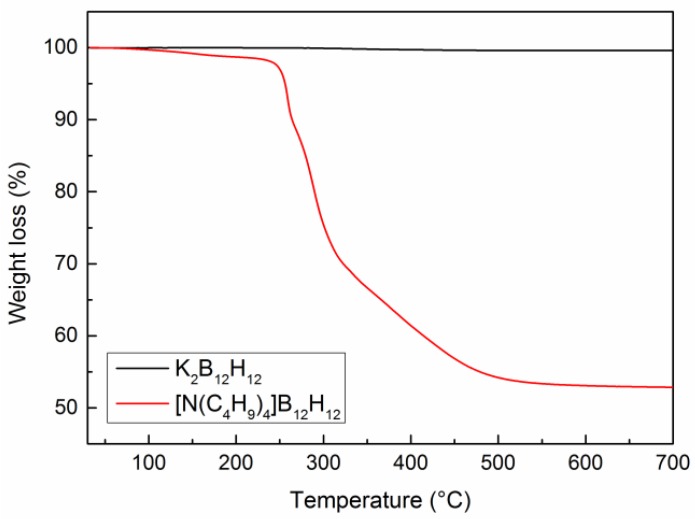
TGA analysis of potassium dodecahydrododecaborate (K_2_B_12_H_12_) and bis-tetrabutyl ammonium *closo*-dodecahydrododecaborate [N(C_4_H_9_)_4_]_2_B_12_H_12_.

The thermal stability of IPs was investigated by means of TGA. Figure 3 illustrates that no weight loss occurred below 100 °C for both IPs [K_2_B_12_H_12_ and N(C_4_H_9_)_4_B_12_H_12_], which indicates the absence of residual solvents. K_2_B_12_H_12_ shows no weight loss and remains stable up to the final temperature of 700 °C. For comparison, sample [N(C_4_H_9_)_4_]B_12_H_12_ shows a large weight loss (~45%) between 200–500 °C. In this temperature range, [N(C_4_H_9_)_4_]B_12_H_12_ decomposes into gaseous products. From these results, we conclude that the K_2_B_12_H_12_ are thermally stable up to 700 °C. This is relevant for the preparation of MMMs, since heating the polymer matrix above the *T_g_* or *T_m_* can reduce the formation of non-selective voids [34].

**Figure 4:**
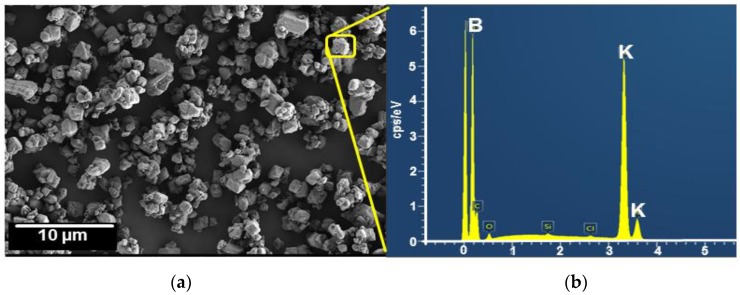
SEM image (**a**) and EDX spectra (**b**) of K_2_B_12_H_12_.

Figure 4a shows the SEM image of K_2_B_12_H_12_ with a distinct crystalline structure. The chemical composition of the IPs was analyzed by EDX spectrometer (Carl Zeiss AG, Oberkochen, Germany), which was attached to the SEM image (Figure 4b). The EDX spectra clearly shows the strong signal of potassium (K) and boron (B) in the crystalline structure of K_2_B_12_H_12_. 

### 5.2. Mixed Matrix Membranes (MMMs) Characterization

**Figure 5:**
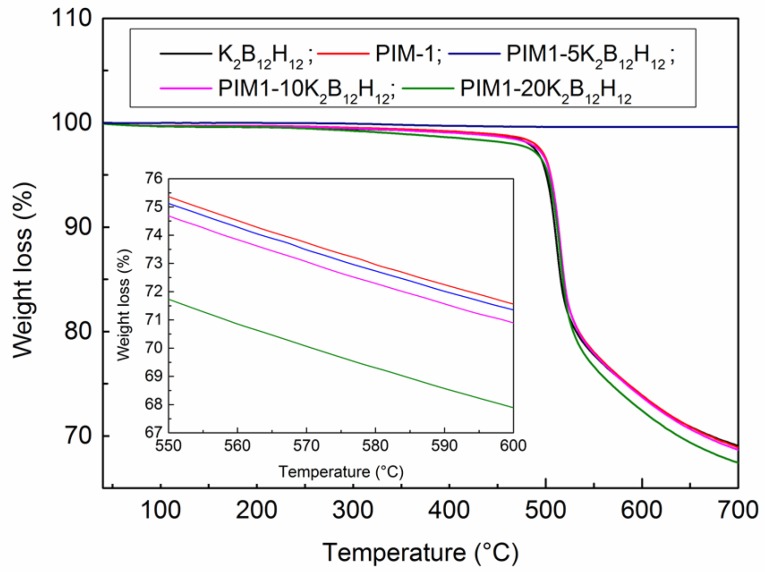
TGA analysis of the pure PIM-1 and PIM1/K_2_B_12_H_12_ MMMs.

The effect of temperature on the degradation of pristine PIM-1 and PIM1/K_2_B_12_H_12_ MMMs at a various loading of K_2_B_12_H_12_ is shown in Figure 5. TGA analysis suggests that no residual solvent was present in the films. The PIM1/K_2_B_12_H_12_ MMMs with 5, 10 and 20 wt % loading of K_2_B_12_H_12_ show similar decomposition stages compared to pure PIM-1 and the onset degradation temperature of these samples was observed at 501 ± 2 °C. The higher magnification TGA results of PIM1/K_2_B_12_H_12_ MMMs from 550 to 600 °C were shown the inset Figure 5. Due to the lack of rotational mobility in the backbone of the rigid ladder polymer, it is difficult to observe a glass transition before the degradation of pristine PIM-1 and its MMMs [16]. 

Table 1 shows the density and weight loss of various wt % of K_2_B_12_H_12_ in PIM1/K_2_B_12_H_12_ MMMs up to a temperature 650 °C. During TGA analysis, the initial weight loss of the samples was affected by buoyancy, which means that the samples and ceramic pan appeared to gain weight before significant decomposition occurred due to the difference in thermal conductivity, density and heat capacity for the purging gas and the sample [35]. However, the buoyancy effect was less apparent at a higher temperature. Thus, the initial wt % of all the samples was set at 100 °C. The K_2_B_12_H_12_ concentration in the polymer was considered as the volume fraction (*φ_IP_*), which appears slightly higher than the weight fraction term due to the density difference between K_2_B_12_H_12_ and polymer. PIM-1 and PIM1/K_2_B_12_H_12_ MMMs began weight loss at approximately 501 ± 2 °C. The weight loss up to 700 °C (*W*_700_) increased slightly with the addition of K_2_B_12_H_12_.

**Figure 6:**
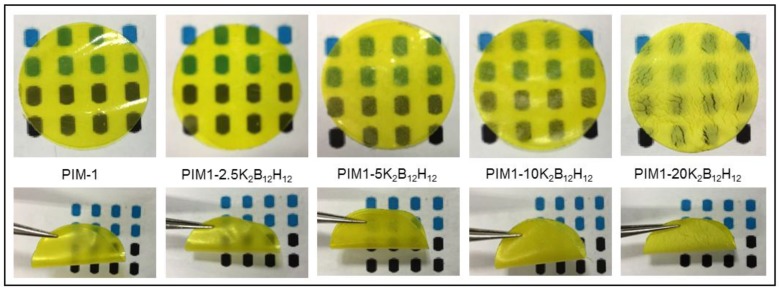
Optical images of PIM-1 and PIM1/K_2_B_12_H_12_ MMMs.

The optical transparencies of PIM-1 and PIM1/K_2_B_12_H_12_ MMMs are shown in Figure 6. These images confirm the improved dispersion of the inorganic particles up to 10 wt % loading. At higher filler content (20 wt %), there is greater agglomeration of inorganic particles in the polymer matrix (see PIM-20 K_2_B_12_H_12_ MMMs film in Figure 6). PIM-1 and PIM1/K_2_B_12_H_12_ MMMs films were more flexible and mechanically stable. The mechanical stability deteriorated beyond 20 wt % filler content in the polymer. 

**Figure 7:**
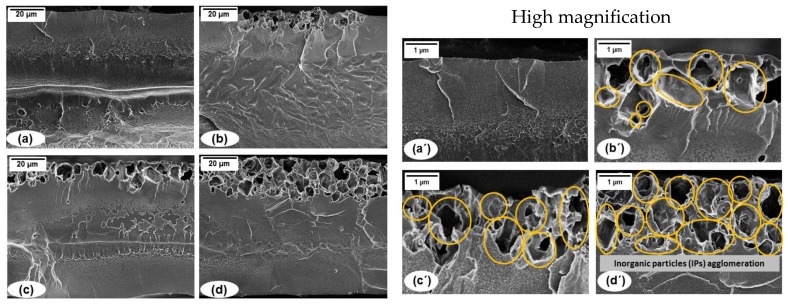
Cross section SEM images of (**a**,**a′**) PIM-1, PIM1/K_2_B_12_H_12_ MMMs incorporated with (**b**,**b′**) 5 wt %, (**c**,**c′**) 10 wt % and (**d**,**d′**) 20 wt % of K_2_B_12_H_12_.

Figure 7 shows the cross-sectional SEM images of PIM-1 and PIM1/K_2_B_12_H_12_ MMMs at different K_2_B_12_H_12_ loadings. K_2_B_12_H_12_ tend to be well-distributed throughout the membrane surface with a 5 and 10 wt % K_2_B_12_H_12_ loading. (Figure 7b,c). As the K_2_B_12_H_12_ loadings were further increased to 20 wt %, the K_2_B_12_H_12_ started to agglomerate throughout the PIM-1 matrix (see Figure 6). Figure 7a–d shows highly-magnified images of the PIM-1 and PIM1/K_2_B_12_H_12_ MMMs incorporated with 5, 10 and 20 wt % of K_2_B_12_H_12_ (showed by a yellow circle). From this observation and optical images (see Figure 6), we can conclude that the threshold limit for the addition of K_2_B_12_H_12_ into the polymer matrix to prevent agglomeration is typically around 20 wt % and the optimum for the addition of K_2_B_12_H_12_ is lower than 20 wt %. 

### 5.3. Gas Permeation Properties

#### 5.3.1. Effects of K_2_B_12_H_12_ Content on PIM1/K_2_B_12_H_12_ MMM Gas Separation Performance

In order to systematically study the effect of K_2_B_12_H_12_ loading on the PIM1/K_2_B_12_H_12_ MMM gas separation performance, MMMs were fabricated with different wt % incorporation of K_2_B_12_H_12_. The permeability results of PIM1/K_2_B_12_H_12_ MMMs for H_2_, O_2_, N_2_, CO_2_ and CH_4_ gases are shown in Table 2. The order of gas permeability was observed as CO_2_ > H_2_ > O_2_ > CH_4_ > N_2_. The addition of 2.5 wt % of K_2_B_12_H_12_ loading to the polymer matrix resulted in a 3% increase in the permeability of H_2_, while the permeability of N_2_, O_2_, CO_2_ and CH_4_ increased 16%, 10%, 17%, and 23%, respectively. Furthermore, a significant enhancement in permeability as a function of K_2_B_12_H_12_ loading in the polymer matrix was observed between 5 to 10 wt %. From the previous report on the permeation enhancement of MMMs [5], these results suggest that the interaction between polymer-chain segments and IPs may disrupt the polymer-chain packing and thus enhance the gas diffusion due to more free volume introduced among the polymer chains and defects at the polymer/IP interface. The permeability of gas molecules such as H_2_, N_2_, O_2_, CO_2_ and CH_4_ decreases as K_2_B_12_H_12_ loading increased from 10 to 20 wt % in the polymer matrix. Some agglomerates form in the polymer matrix at high loading (20 wt %), which may decrease the total free volume and tortuosity around the agglomerated K_2_B_12_H_12_ domains, leading to a slight deterioration of the permeation.

**Figure 8:**
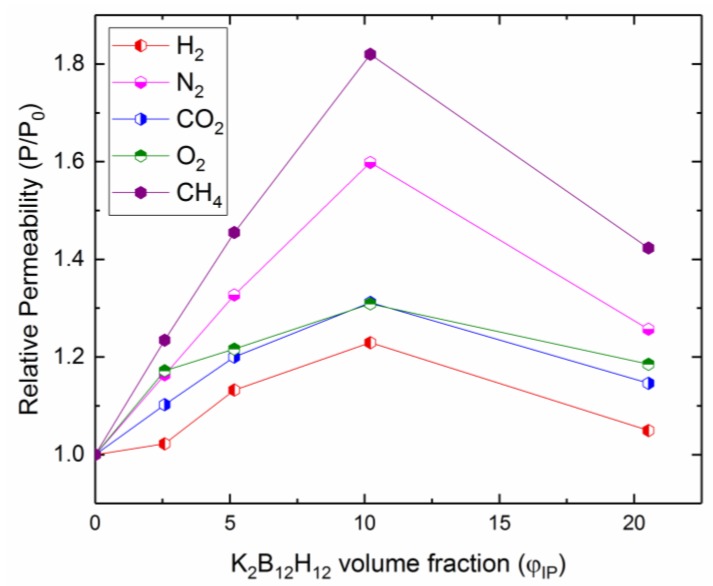
Relative permeability (i.e., ratio of permeability of PIM1/K_2_B_12_H_12_ with pure polymer PIM-1) of PIM1/K_2_B_12_H_12_ MMMs to a variety of gas penetrates as a function of K_2_B_12_H_12_ volume fraction (*φ_IP_*).

In addition, the gas permeabilities of PIM-1 containing K_2_B_12_H_12_ were higher than pure PIM-1 and increasing up to the optimum limit. This trend is clearly depicted in Figure 8, which presents the normalized permeability of PIM1/K_2_B_12_H_12_ MMMs for O_2_, N_2_, CH_4_, and CO_2_ gases as a function of K_2_B_12_H_12_ volume fraction (*φ_IP_*). 

Table 3 shows the ideal separation factors for pure PIM-1 and PIM1/K_2_B_12_H_12_ MMMs. At 2.5–10 wt % K_2_B_12_H_12_ loading, the permselectivity was found to be decreased compared to the pure PIM-1. However, the selectivity increased at 20 wt % K_2_B_12_H_12_ loading due to a significant decrease in permeability. It is shown in Table 3 that the CH_4_/N_2_ separation factor increased as the amount of K_2_B_12_H_12_ increased due to the higher adsorption capacity for CH_4_ over N_2_. Despite increases in the permeability of O_2_ and N_2_, the O_2_/N_2_ separation factor remained virtually unchanged because K_2_B_12_H_12_ were not selective for either O_2_ or N_2_. In addition, the constant O_2_/N_2_ separation factor in PIM1/K_2_B_12_H_12_ MMMs suggests that the prepared membranes do not have any unselective voids at the polymer/K_2_B_12_H_12_ interface.

**Figure 9:**
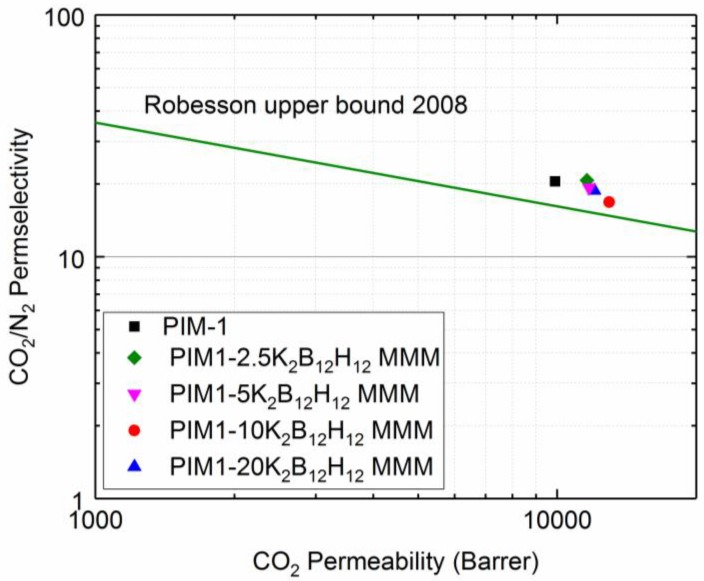
Trade-off between CO_2_ permeability and CO_2_/N_2_ permselectivity of PIM-1 and PIM1/K_2_B_12_H_12_ MMMs relative to Robeson upper bound plot.

Recently, various trends of MMMs in terms of relative trade-off in permeability and permselectivity have been noted. Many permselectivity increments were seen with the addition of activated carbon, fused silica and metal organic frameworks (MOF) [36]. The gas separation performance of PIM1/K_2_B_12_H_12_ MMMs was plotted on a Robeson upper bound plot in order to compare the results with the literature data. The Figure 9 shows the Robeson upper bound 2008 [37] for CO_2_/N_2_ gas pairs and the results of these MMMs with different filler content. The incorporation of fillers in the PIM-1 polymer increases the efficiency of this membrane type in the separation of CO_2_ gas over N_2_.

#### 5.3.2. Influence of Temperature on the Gas Separation Performance of PIM1/K_2_B_12_H_12_ MMMs

**Figure 10:**
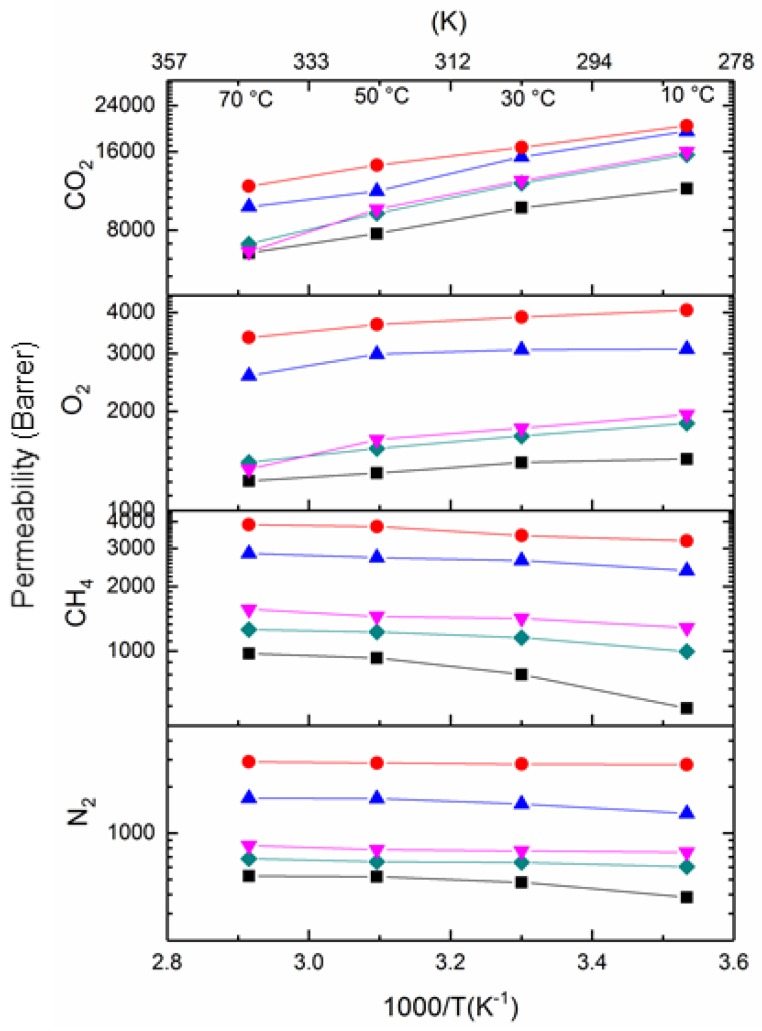
Permeability of N_2_, CH_4_, CO_2_ and O_2_ in PIM-1 and PIM1/K_2_B_12_H_12_ MMMs as a function of reciprocal temperature ((■-black) PIM-1, (♦-olive) 2.5 wt % PIM1/K_2_B_12_H_12_ MMM, (▼-pink) 5 wt % PIM1/K_2_B_12_H_12_ MMM, (●-red) 10 wt % PIM1/K_2_B_12_H_12_ MMM, (▲-blue) 20 wt % PIM1/K_2_B_12_H_12_ MMM).

Temperature effects on PIM1/K_2_B_12_H_12_ MMMs were studied over a temperature range of 283–343 K (10, 30, 50 and 70 °C) for single gas at one bar feed pressure. Figure 10 shows the permeability of N_2_, CH_4_, CO_2_, and O_2_ for PIM-1 and PIM1/K_2_B_12_H_12_ MMMs as a function of the inverse absolute temperature. From Figure 10, it can be seen that the permeability of N_2_ and CH_4_ increased with increasing temperature, while for CO_2_ and O_2_, the permeability decreased with increasing temperature. This result indicates that highly sorbed gases like CO_2_ do not affect the permeation rate of lighter gases in subsequent runs [15,38]. However, a careful examination shows that the permeability of all gases is higher in 2.5–10 wt % than 20 wt % PIM1/K_2_B_12_H_12_ MMMs and the pristine PIM-1 membrane at each temperature.

**Figure 11:**
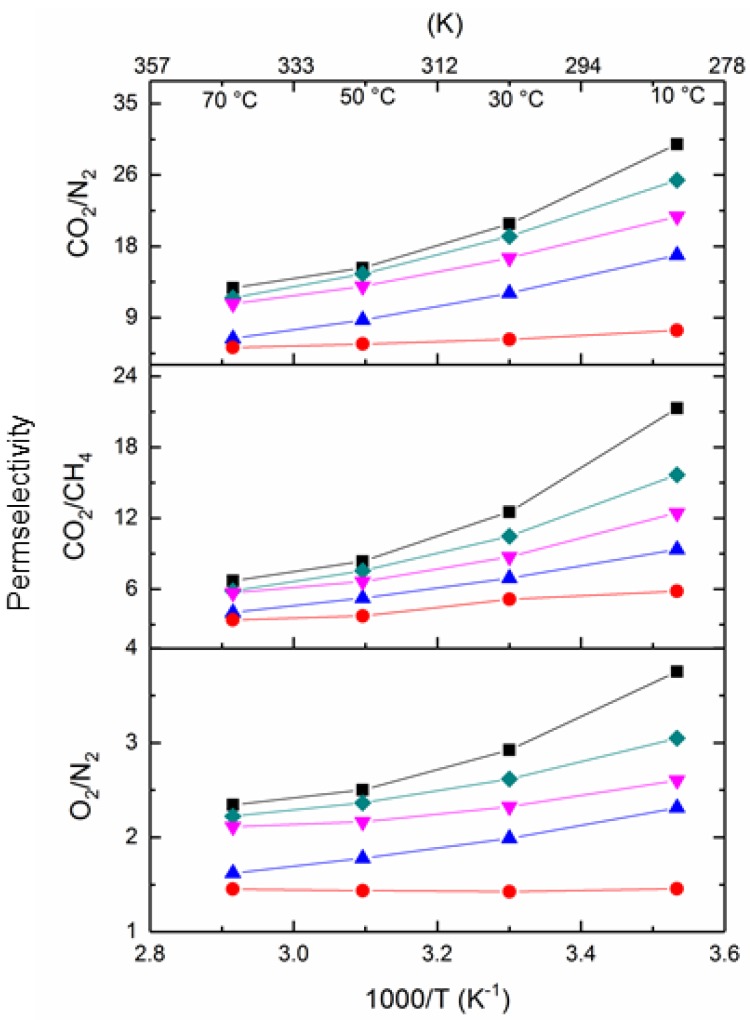
Selectivity of O_2_/N_2_, CO_2_/N_2_ and CO_2_/CH_4_ in PIM-1 and PIM1/K_2_B_12_H_12_ MMMs as a function of reciprocal temperature ((■-black) PIM-1, (♦-olive) 2.5 wt % PIM1/K_2_B_12_H_12_ MMM, (▼-pink) 5 wt % PIM1/K_2_B_12_H_12_ MMM, (●-red) 10 wt % PIM1/K_2_B_12_H_12_ MMM, (▲-blue) 20 wt % PIM1/K_2_B_12_H_12_ MMM).

Figure 11 shows the O_2_/N_2_, CO_2_/N_2_ and CO_2_/CH_4_ selectivity of the pure PIM-1 and PIM1/K_2_B_12_H_12_ MMMs as a function of the inverse of absolute temperature. It was observed that the selectivity for a given gas pair decreases with an increase in the temperature of pure PIM-1 and PIM1/K_2_B_12_H_12_ MMMs. Hence, the incorporation of K_2_B_12_H_12_ does not change any selectivity pattern at a higher temperature. However, a significant difference in selectivity at a lower temperature was observed for PIM-1. It shows that the O_2_/N_2_ selectivity at 333 K is nearly 2.7; at 283 K it reaches 4.6, while for CO_2_/N_2_ selectivity is around 30.9 at low temperature and 14.1 at elevated temperature 343 K (70 °C).

In order to understand the temperature dependence of N_2_, O_2_, CO_2_ and CH_4_ permeabilities in PIM1/K_2_B_12_H_12_ MMMs, the data were correlated with the Arrhenius equation and the activation energy of permeation (*E_P_*) was determined using the following relationship:

(13) $$P=P_{0\;}exp\left(\frac{-E_{P}}{RT}\right)$$

where *P* is the gas permeability, *P*_0_ is the pre-exponential factor, (*E_P_*) is the activation energy of permeation (J/mol), *R* is the gas constant (8.314 J/(mol·K)) and *T* is the absolute temperature. The given equation was valid in a temperature range that does not cause significant thermal transitions in the polymer. Table 4 shows the activation energy of permeation (*E_P_*) of PIM-1 and PIM1/K_2_B_12_H_12_ MMMs, which were determined from the slope of the Arrhenius plot. 

According to the literature, the activation energy of permeation was the sum of the activation energy of diffusion (*E_D_*), and the enthalpy of sorption (*ΔH_S_*),

(14) $$E_{P}=E_{D}+\;\Delta H_{S}$$

Generally, the gas permeability of all conventional glassy polymers increases with increased temperature, because *E_D_ + H_S_* > 0 and |*E_D_*|/|*H_S_*| > 1. An exception to this rule is the temperature dependence of gas permeability in high free volume polymers such as PIM-1, i.e., gas permeabilities decrease with increase temperature for condensable gas (e.g., CO_2_), where |*E_D_*|/|*H_S_*| < 1 [15]. Therefore, the negative activation energies of permeation in PIM-1 and PIM1/K_2_B_12_H_12_ MMMs result from very small activation energies of diffusion, which indicates that the dependence of permeability on temperature is much weaker. In addition, the negative value of *E_P_* is characteristic of the decrease of CO_2_ permeability with the increase of temperature, which was clearly observed in Figure 10. Another case, the N_2_ permeability of PIM-1 and PIM1/K_2_B_12_H_12_ MMMs, was strongly temperature-dependent and *E_P_* values were the same order of magnitude as those of conventional glassy polymers. Moreover, negative *E_P_* was observed for microporous solids in which the pore dimensions were relatively larger than the diffusing gas molecules [39].

### 5.4. Gas Sorption

#### 5.4.1. Static Gas Sorption

**Figure 12:**
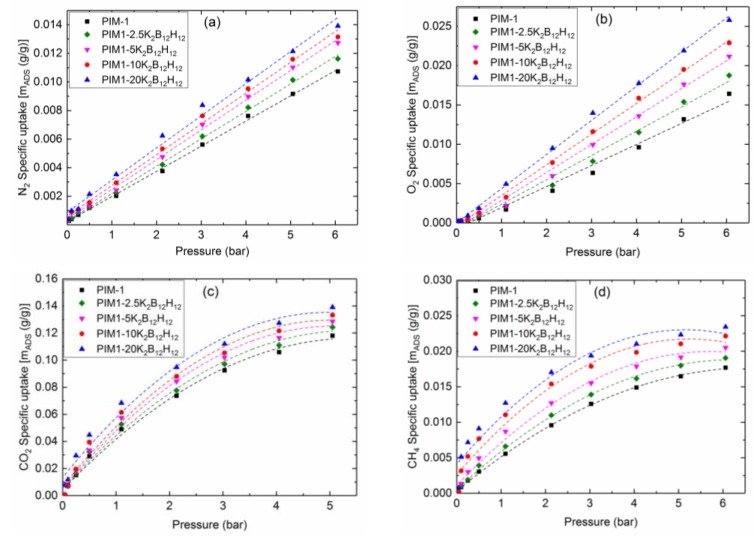
(**a**) N_2_; (**b**) O_2_; (**c**) CO_2_; and (**d**) CH_4_ adsorption isotherm in PIM-1 and PIM1/K_2_B_12_H_12_ MMMs (dashed lines represent the fitting curve).

Static gas sorption measurements were performed to characterize the sorption behavior of pure PIM-1 and PIM1/K_2_B_12_H_12_ MMMs. Figure 12 represents N_2_, O_2_, and CH_4_ sorption isotherms in PIM-1 and PIM-1 containing 2.5, 5, 10 and 20 wt % K_2_B_12_H_12_ at 303 K. From Figure 12, the sorption of N_2_ and O_2_ was much less than that of other gases, such as CO_2_ and CH_4_, owing to their lower condensability and weak interaction with the PIM-1 polymer. On the other hand, the sorption curve concave to the pressure axis was observed for CO_2_ and CH_4_, this was a general trend for glassy polymers and can be described by the so-called dual-mode sorption model [40,41]. The amount of gas absorbed in PIM1/K_2_B_12_H_12_ MMMs films for each gas depends on the K_2_B_12_H_12_ content, as shown in Figure 12. The relative increase in gas absorption was small at 20 wt % of K_2_B_12_H_12_ loading (showing more discrepancy in sorption measurement—see Figure 12) in comparison to the increases seen at 2.5, 5 and 10 wt % of K_2_B_12_H_12_ loading. Therefore, the presence of K_2_B_12_H_12_ increases the relative sorption of gases in the membrane, at higher K_2_B_12_H_12_ contents; this increase could be constrained polymer chain packing at the K_2_B_12_H_12_/polymer interface.

When both sorption isotherms of CO_2_ and CH_4_ were fitted with the dual-mode sorption model (Equation (2)), Henry′s constants (*k_D_*), the Langmuir capacity constants (*C′_H_*) and the Langmuir affinity constants (*b*) can be obtained using a non-linear regression method and these were shown in Table 5. The low Henry constants for both CO_2_ and CH_4_ indicate that the major sorption mechanism inside PIM-1 was Langmuir sorption, which takes place in the non-equilibrium excess volume occurring in glassy polymers [42]. The addition of IPs to the polymer matrix could affect and possibly disturb or alter this excess volume. When the dual mode sorption parameters for different wt % of K_2_B_12_H_12_ are compared in Table 5, an increase with increasing K_2_B_12_H_12_ loading was visible for all parameters for both CO_2_ and CH_4_. This implies that the addition of K_2_B_12_H_12_ increases the maximum sorption capacity and the affinity towards CO_2_ and CH_4_, but does not provide any additional sorption selectivity, since the ideal sorption selectivity does not increase. 

The maximum sorption capacity, *C′_H_*, in 20 wt % PIM1/K_2_B_12_H_12_ MMMs was decreased by 5% and 2% for CO_2_ and CH_4_, respectively, compared to 10 wt % PIM1/K_2_B_12_H_12_ MMMs. This difference can be explained by sorption limitations in the K_2_B_12_H_12_ particles due to the surrounding polymer. From the SEM images in Figure 7c,c′, there was large area of agglomeration between the polymer matrix and the K_2_B_12_H_12_ at 20 wt % loading, which might reduce the sorption capacity on the outside of the K_2_B_12_H_12_, where interaction with the polymer takes place, or limits the diffusion into K_2_B_12_H_12_. Moreover, the addition of K_2_B_12_H_12_ particles might have an influence on the diffusion coefficient, which is discussed in the next paragraph.

#### 5.4.2. Dynamic Gas Sorption

**Figure 13:**
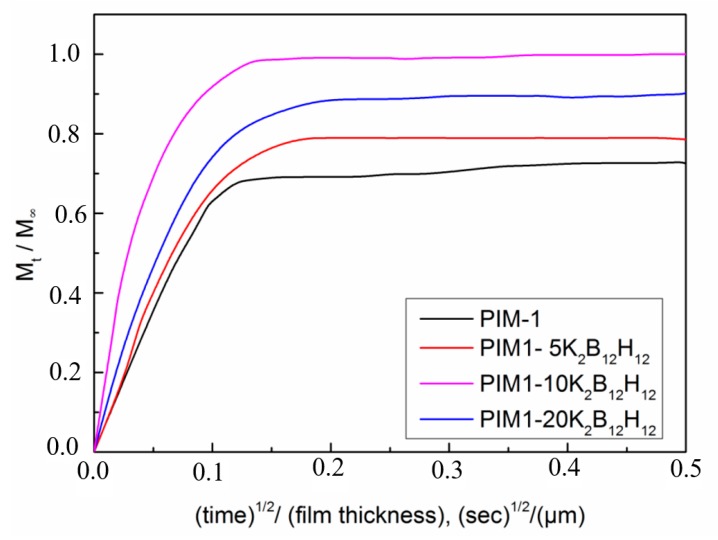
CO_2_ kinetic uptake curves in PIM-1 and PIM1/K_2_B_12_H_12_ MMMs at 303 K and 1 bar.

Dynamic sorption experiments were performed to determine the kinetic diffusion coefficients of the PIM-1 and PIM1/K_2_B_12_H_12_ MMMs with various wt % of K_2_B_12_H_12_. It was important to verify whether all fitting parameters can be accurately obtained with the given film thickness. Figure 13 depicts the CO_2_ kinetic sorption fractional uptake curves in PIM-1 and PIM-1 containing 5 wt %, 10 wt % and 20 wt % K_2_B_12_H_12_. From Figure 13, CO_2_ uptake kinetics was normalized to account for differences in film thickness, and the sorption equilibrium was attained much more rapidly in PIM1/K_2_B_12_H_12_ MMMs than pure PIM-1. This result implies faster diffusion in PIM1/K_2_B_12_H_12_ MMMs, which is qualitatively consistent with the concentration-averaged diffusion coefficients.

In addition to the diffusion coefficient (*D*) that was calculated from steady-state transport data, diffusion coefficients may also be estimated from the dynamic sorption. Kinetic or transient diffusion coefficients, *D*, were extracted from the data in Figure 13 by application of the one-dimensional form of following Equation (15), which was modified from Equation (3) for Fick′s diffusion law:

(15) $$\frac{M_{t}}{M_{\infty }}=4{\left(\frac{Dt}{\pi l^{2}}\right)}^{1/2}$$

where *M_t_* was the mass gain (by the polymer film) at time *t*, *M_∞_* is the maximum mass gain, *D* was the diffusivity gas penetrant and *l* was the thickness of the film. 

**Figure 14:**
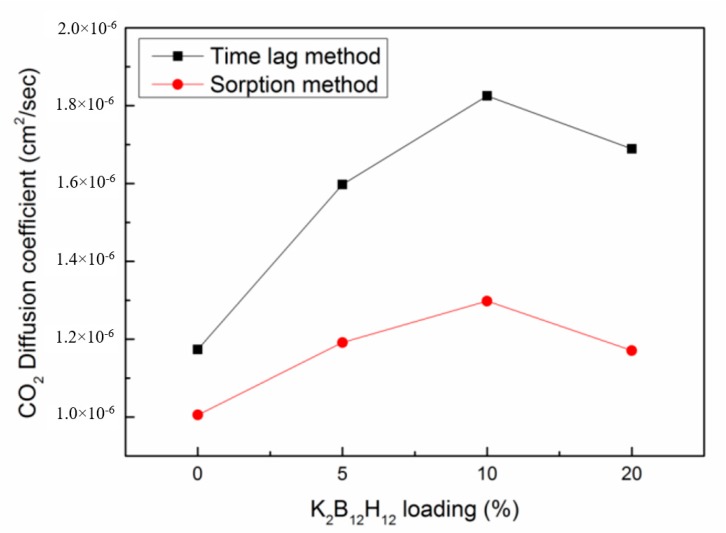
CO_2_ diffusion coefficient in PIM-1 and PIM1/K_2_B_12_H_12_ MMMs.

Figure 14 shows CO_2_ diffusion coefficients in PIM-1 and PIM1/K_2_B_12_H_12_ MMMs determined from the kinetic sorption studies (using Equation (15)). The diffusion coefficients were calculated from the time-lag method, included as well in Figure 14 for comparison. 

Although the absolute values of the diffusion coefficients obtained by the time-lag and kinetic sorption methods were different [43], qualitatively the changes were consistent. Typically, the kinetic diffusion coefficients measured by gravimetric sorption were lower than those obtained by the time-lag. This discrepancy in results was observed because kinetic (transient) uptake experiments involve additional diffusion into the dead-end pores, while transport through dead-end pores does not play a role in steady-state permeation (time-lag) experiments [44]. 

## 6. Conclusions

Mixed matrix membranes were prepared successfully adding different amounts of K_2_B_12_H_12_ as IPs into a PIM-1 as a polymer matrix. The prepared PIM1/K_2_B_12_H_12_ MMMs were characterized by scanning electron microscopy (SEM), thermogravimetric analysis (TGA), single gas permeation tests and sorption measurement. K_2_B_12_H_12_ were well-dispersed in the polymer matrix at a loading of 2.5, 5, and 10 wt %, while at 20 wt % the K_2_B_12_H_12_ forms agglomeration and phase separation in the polymer matrix, which was confirmed by SEM and optical images. The permeability performance of the prepared PIM1/K_2_B_12_H_12_ MMMs mainly depends on the addition of IPs rather than the effect of the interfacial zone because the O_2_/N_2_ gas pair selectivity was constant for all MMMs. Overall increases in gas permeability and diffusivity were observed for all tested gases, suggesting that IPs could disrupt more polymer chain packing. The sorption isotherms in PIM-1 and PIM1/K_2_B_12_H_12_ MMMs exhibited typical dual-mode sorption behaviors for the gases CO_2_ and CH_4_. The CO_2_ diffusion coefficient calculated by the dynamic sorption method was lower than the time-lag method for PIM-1 and PIM1/K_2_B_12_H_12_ MMMs. This is the first report of the gas transport performance of MMMs using K_2_B_12_H_12_ and a PIM-1 polymer. It is clear that the addition of K_2_B_12_H_12_ to a polymer matrix can improve certain gas pair selectivities, as well as the permeability of small gas molecules.

## Reference

## Figures and Tables

**Table 1:** Physical and thermal properties of K_2_B_12_H_12_, PIM-1, and PIM1/K_2_B_12_H_12_ MMMs.

Membrane	Volume Fraction *φ_IP_* (%)	K_2_B_12_H_12_ Loading (%)	*w*_700_ (%)	*ρ* (g/cm^3^)
PIM-1	0	0	32.17	1.066
PIM-2.5 K_2_B_12_H_12_	2.58	2.5	32.46	1.078
PIM-5 K_2_B_12_H_12_	5.16	5	33.10	1.077
PIM-10 K_2_B_12_H_12_	10.21	10	33.20	1.072
PIM-20 K_2_B_12_H_12_	20.53	20	34.43	1.067
K_2_B_12_H_12_	-	-	0.6	1.031 *

* determined from Micromeritics AccuPyc 1330 pycnometer. *w*_700_: weight loss up to 700 °C. *ρ*: density of membrane.

**Table 2:** Gas permeabilities of various gases in pure PIM-1 and PIM1/K_2_B_12_H_12_ MMMs.

Membrane	Permeability (Barrer)				
	H_2_	N_2_	O_2_	CO_2_	CH_4_
PIM-1	3274 ± 5	483 ± 10	1396 ± 13	9896 ± 28	789 ± 15
PIM1-2.5 K_2_B_12_H_12_ MMM	3347 ± 8 (3%)	562 ± 11 (16%)	1539 ± 12 (10%)	11598 ± 20 (17%)	974 ± 18 (23%)
PIM1-5 K_2_B_12_H_12_ MMM	3707 ± 9 (13%)	641 ± 10 (33%)	1675 ± 11 (20%)	12036 ± 21 (22%)	1148 ± 16 (45%)
PIM1-10 K_2_B_12_H_12_ MMM	4025 ± 8 (22%)	772 ± 9 (60%)	1831 ± 14 (31%)	12954 ± 23 (31%)	1436 ± 16 (82%)
PIM1-20 K_2_B_12_H_12_ MMM	3436 ± 7 (5%)	607 ± 12 (25%)	1600 ± 14 (14%)	11729 ± 23 (18%)	1123 ± 14 (42%)

(% increment from pure polymer).

**Table 3:** Selectivity of various gas pairs for pure PIM-1 and PIM1/K_2_B_12_H_12_ MMMs.

Membrane	Permselectivity					
	H_2_/N_2_	H_2_/CH_4_	CH_4_/N_2_	O_2_/N_2_	CO_2_/N_2_	CO_2_/CH_4_
PIM-1	6.8	4.2	1.6	2.9	20.5	12.5
PIM1-2.5 K_2_B_12_H_12_ MMM	6.0	3.4	1.7	2.7	20.7	11.9
PIM1-5 K_2_B_12_H_12_ MMM	5.8	3.2	1.8	2.6	18.8	10.5
PIM1-10 K_2_B_12_H_12_ MMM	5.2	2.8	1.9	2.4	16.8	9.0
PIM1-20 K_2_B_12_H_12_ MMM	5.6	3.0	1.8	2.6	19.3	10.4

**Table 4:** Activation energy of permeation for pristine PIM-1 and PIM1/K_2_B_12_H_12_ MMMs.

Membrane	E_P_ (kJ/mol)	
	N_2_	CO_2_
PIM-1	18.5	−3.3
PIM1-2.5 K_2_B_12_H_12_ MMM	13.7	−4.0
PIM1-5 K_2_B_12_H_12_ MMM	6.4	−4.6
PIM1-10 K_2_B_12_H_12_ MMM	5.5	−5.0
PIM1-20 K_2_B_12_H_12_ MMM	2.4	−3.1

**Table 5:** Fitted dual-mode sorption parameters in PIM-1 and PIM1/K_2_B_12_H_12_ MMMs of CO_2_ and CH_4_ sorption isotherm.

Feed Gas	K_2_B_12_H_12_ Loading (wt %)	Dual Mode Sorption Model Parameter		
		*k_D_*	*C′_H_*	*b*
CO_2_	0	2.330	104.630	0.415
	2.5	2.440	105.830	0.422
	5	2.530	113.750	0.440
	10	2.600	120.105	0.491
	20	2.58	115.81	0.444
CH_4_	0	0.581	62.097	0.135
	2.5	0.59	63.957	0.141
	5	0.604	65.042	0.154
	10	0.627	66.741	0.167
	20	0.611	65.412	0.151

Units of *k_D_* = cm^3^(STP)/(cm^3^·atm)_polymer_*, C′_H_ =* cm^3^(STP)/cm^3^_polymer_, *b* = atm^−1^.
